# Biochemistry, Safety, Pharmacological Activities, and Clinical Applications of Turmeric: A Mechanistic Review

**DOI:** 10.1155/2020/7656919

**Published:** 2020-05-10

**Authors:** Rabia Shabir Ahmad, Muhammad Bilal Hussain, Muhammad Tauseef Sultan, Muhammad Sajid Arshad, Marwa Waheed, Mohammad Ali Shariati, Sergey Plygun, Mohammad Hashem Hashempur

**Affiliations:** ^1^Institute of Home and Food Science, Government College University, Faisalabad 38000, Pakistan; ^2^BZU, Multan 66000, Pakistan; ^3^Laboratory of Biocontrol and Antimicrobial Resistance, Orel State University Named after I.S. Turgenev, Orel 302026, Russia; ^4^European Society of Clinical Microbiology and Infectious Diseases, Basel 4051, Switzerland; ^5^All Russian Research Institute of Phytopathology, Moscow 143050, Russia; ^6^Noncommunicable Diseases Research Center, Fasa University of Medical Sciences, Fasa, Iran; ^7^Department of Traditional Persian Medicine, Fasa University of Medical Sciences, Fasa, Iran

## Abstract

Turmeric (*Curcuma longa* L.) is a popular natural drug, traditionally used for the treatment of a wide range of diseases. Its root, as its most popular part used for medicinal purposes, contains different types of phytochemicals and minerals. This review summarizes what is currently known on biochemistry, safety, pharmacological activities (mechanistically), and clinical applications of turmeric. In short, curcumin is considered as the fundamental constituent in ground turmeric rhizome. Turmeric possesses several biological activities including anti-inflammatory, antioxidant, anticancer, antimutagenic, antimicrobial, antiobesity, hypolipidemic, cardioprotective, and neuroprotective effects. These reported pharmacologic activities make turmeric an important option for further clinical research. Also, there is a discussion on its safety and toxicity.

## 1. Introduction

Functional foods have been used traditionally for medicinal purposes through history [1, 2]. In recent decades, there is an increasing interest in research on functional foods and dietary supplements for different diseases [3–6]. Turmeric is one of the most popularly investigated functional foods [7]. Turmeric (*Curcuma longa* L.; syn.: *Curcuma domestica* Valeton) belongs to the Zingiberaceae family and is extensively cultured in the tropical areas of Asia [8]. Alternative names frequently used for turmeric are turmeric root and yellow root. It generally attains a height of 3–5 feet and has oblong leaves with yellowish funnel-shaped flowers. *C. longa* can be grown in diverse environmental situations at a temperature of 20–35°C with yearly rain of 1500 mm. It grows in well-drained sandy or clay loam soils, having a pH of 4.5–7.5 with good organic status, where it flourishes outstandingly [9].

**Figure 1:**
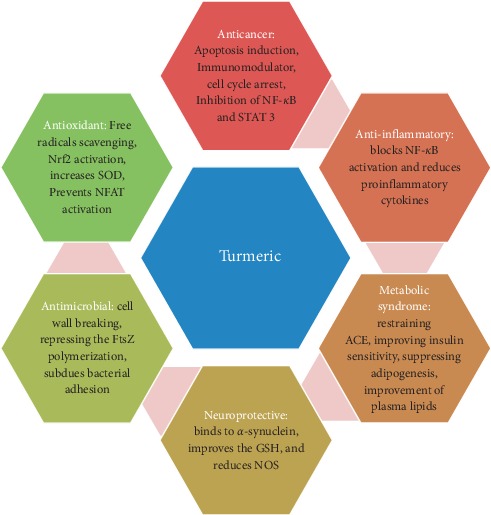
A schematic diagram representing turmeric's medicinal attributes regarding some of the most important mechanisms. NF-*κ*B: nuclear factor-*κ*B; STAT3: signal transducer and activator of transcription 3; Nrf2: nuclear factor erythroid 2-related factor 2; SOD: superoxide dismutase; NFAT: nuclear factor of activated T cells; FtsZ: filamenting temperature-sensitive mutant Z; GSH: glutathione; NOS: nitric oxide synthase; ACE: angiotensin-converting enzyme.

Turmeric has a long antiquity for its use as a spice in cuisines of Asian countries and also in other areas, globally. For example, it is known as *Zard choobe* in Persian. It enhances the flavor and improves the color tonality of foods like rice, yogurt, and chicken. However, consumers prefer to use it with other spices to enrich the flavor. Several communities across the globe use turmeric and its variant fractions to formulate certain traditional medications to cure human ailments with especial reference to China, India, Iran, and Indonesia. Turmeric has long been utilized as a tonic. It is also used for a wide variety of diseases including dyslipidemia, stomach disorders, arthritis, and hepatic diseases [10–12]. Curcumin (1,7-bis(4-hydroxy-3-methoxyphenyl)-1E,6E-heptadiene-3,5-dione or diferuloyl methane) is a polyphenol derived from the turmeric. Curcumin is a yellow tincture, which can be obtained from the rhizome of the plant [13]. Yellow color of turmeric is owing to the existence of curcumin, which consists of three main curcuminoid complexes: curcumin I, curcumin II, and curcumin III [14]. The dehydrated root portion of turmeric holds up to 8% curcumin [15]. Curcumin is insoluble in water and ether, but it has the ability to disperse in ethanol and other organic solvents [16]. The diferuloylmethane and volatile oils are other main active ingredients of turmeric. Turmeric has been mentioned to have numerous biological effects including anti-inflammatory, antimicrobial and antitumoral activity, antioxidant, and hypolipidemic properties [17–21]. Furthermore, turmeric has also been reported as a protective agent against various cancers.  Figure 1 represents a schematic view about turmeric's medicinal attributes regarding some of the most important mechanisms. It has been proved that curcumin (as the main active ingredient of turmeric) is a potent natural remedy against the process of inflammation and oxidation, thus making it possible to be used as a protective tool for cancer prevention [22–27]. Moreover, turmeric oil contains essential fatty acids showing antifungal, antimutagenic, and antibacterial activities [28, 29].

In the current review, nutritional value, the most used products of turmeric, and its biochemistry are briefly discussed. Moreover, there was an attempt not only to highlight the anticancer potential of the turmeric and its components but also to discuss other pharmacological activities by mechanistic details, clinical applications, and safety of turmeric.

## 2. Nutritional Composition

Turmeric is a rich source of carbohydrates and fiber. Also, it contains some proteins and fats, but there is no cholesterol in it. Furthermore, it contains pyridoxine, vitamin C, potassium, calcium, magnesium, and phosphorous in appropriate amounts, making it one of the nutritionally rich natural food products.  Table 1 displays the brief nutritional composition of turmeric [30].

## 3. Main Products

Table 2 briefly shows the widely used products of turmeric and their brief descriptions and uses in daily life [31].

## 4. Molecular Constituents

**Figure 2:**
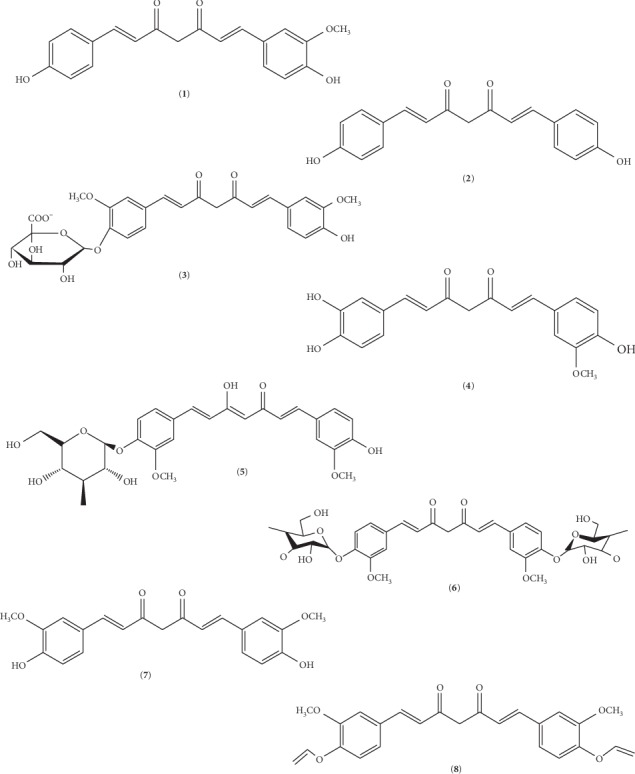
2D molecular structures of curcumin: (1) demethoxycurcumin, (2) bisdemethoxycurcumin, (3) curcumin glucuronide, (4) monodemethylcurcumin, (5) curcumin monoglucoside, (6) curcumin diglucoside, (7) Keto-curcumin, and (8) allyl curcumin (obtained and modified from Open Chemistry Database, National Center for Biotechnology Information, and PubChem Substance Database; https://pubchem.ncbi.nlm.nih.gov/substance).

Turmeric has numerous molecular constituents, each possessing a variety of biological activities. For instance, there are a minimum of 20 molecules that are antibiotic and 14 of its constituents have known cancer preventive activity. Also, 12 of its molecules are antitumor, and the other 12 molecules have anti-inflammatory effects. It contains at least 10 molecular constituents with antioxidant properties, too. Overall, 326 biological activities of turmeric are identified. Three of the constituents that are widely researched in turmeric are gold-colored alkaloids curcuminoids, namely, curcumin, bisdemethoxycurcumin, and demethoxycurcumin.  Figure 2 shows the natural metabolites of curcumin.

## 5. Anticancer Perspectives

In prehistoric times, *C. longa* has been utilized for the management of numerous illnesses [32, 33]. Turmeric and its ingredient can be considered as multitargeted phytochemicals for cancer treatment. For example, apoptosis, autophagy, and cell cycle arrest can be affected by their use [34]. There are so many signaling pathways (e.g., p53, Ras, phosphoinositide 3- kinase, AKT, Wnt/*β*-catenin, and mammalian target of rapamycin) that are anticancer targets of curcumin [35, 36]. Also, regulation of microRNAs network expression is modified by turmeric [37]. It should be noted that histone deacetylases activity is inhibited by curcumin, according to in vitro and in vivo studies [38].

### 5.1. Colorectal Cancer

In recent times, colorectal cancer (CRC) has become an alarming universal health care issue. Data show that obesity and its associated metabolic troubles are linked with colorectal carcinogenesis. Numerous biological mechanisms on the relationship between obesity and the progression of CRC have been established. Insulin resistance and alteration in the insulin-like growth factor-1 (IGF-1) contribute to obesity-related colorectal carcinogenesis [39, 40]. It is also noted that the level of tumor necrosis factor-*α* (TNF-*α*) increases in adipose tissue, which is associated with stimulation of tumor endorsement and development of carcinogenesis [41]. Adipocytokine disproportion and chronic inflammation related to obesity increase the chances of CRC. Curcumin might be an expedient remedy in the prevention of CRC in obese individuals. In fact, it stimulates AMP-activated kinase by lessening the appearance of COX-2 protein and represses the nuclear factor-*κ*B (NF-*κ*B) action on the mucosa of the colon. Curcumin also diminishes the leptin concentration in the serum which conversely increases the adiponectin level [42]. According to another study, poloxamer 407 can be used as a polymer for the expansion of colorectal medication liberation system for curcuminoids in CRC treatment [43].

Turmeric performs antitumor and anticancer functions through the inhibition of NF-*κ*B establishment and downregulation of NF-*κ*B-related gene products associated with endurance, propagation, and metastasis of cancer cells. The spice censors the commencement of signal transducer and activator of transcription 3 (STAT3) and stimulates the death receptors. Also, turmeric improves the creation of reactive oxygen species (ROS) and decreases the expansion of tumor cell lines. Furthermore, turmeric increases the sensitivity of the tumor cells to capecitabine and taxol (chemotherapeutic drugs). It also subdues NF-*κ*B activation initiated by receptor activator of nuclear factor-kappa B ligand (RANKL), which may be associated with the repression of osteoclastogenesis. Hence, turmeric can efficiently obstruct the proliferation of tumor cells by the suppression of NF-*κ*B and STAT3 pathways [44–46]. Furthermore, turmeric can succeed in dealing with the challenge of P-glycoprotein-mediated multidrug resistance of CRC, as exhibited in vitro and in vivo [47].

### 5.2. Renal Cancer

Long-run exposure of a human kidney cell line to 10 *μ*M curcumin changes the swelling-activated chloride current in a dose-dependent way. Curcumin application induces apoptosis in the human kidney cells and stimulates the emergence of a subpopulation of the cells with amplified volume at a concentration of 5.0–10 *μ*M. Likewise, 50 *μ*M curcumin initiates apoptosis and enlarges the size of colorectal adenocarcinoma cells. The cell cycle arrest might be the reason that increases the size of the cell line after exposure to curcumin [48].

### 5.3. Hepatic Cancer

In another research, Yu and colleagues studied the molecular mechanisms of apoptosis induction in human hepatoma SMMC-7721 cells. They reported that curcumin prevents the growth of SMMC-7721 cells appreciably by inducing the apoptosis through the modulation of bax/bcl-2 [49]. It seems that curcumin targets the spindle assembly checkpoint to initiate apoptosis in the cells having higher concentration of phosphorylated cell division cycle 27 (CDC27). Phosphorylation of CDC27 is actually the mechanism by which curcumin exerts its anticancer effect. Curcumin causes cell death by stimulating apoptotic pathway and inhibiting the cell growth and proliferation [50].

### 5.4. Bone Cancer

Dennis and coworkers demonstrated a novel skylight in amalgamation treatment by exploiting a synthetic analogue of natural compound pancratistatin with the curcumin, for the management of osteosarcoma [51]. Although curcumin has strong antiproliferative and anti-inflammatory properties, its low water solubility limits its uses. One controlled study described the preparation and characterization of nanocurcumin using poly-lactic-co-glycolic acid. It seems that water solubility and antitumor activity of the mentioned nanoparticulate formulation significantly improved [52].

### 5.5. Lung Cancer

*C. longa* is presently labeled to own tumor inhibiting gears not only in vitro but also in vivo. It has been testified that curcumin can progress the tumor hindering efficiency of docetaxel in lung cancer. Likewise, synchronized administration of curcumin and docetaxel results in slight toxicity to normal tissues as well as the bone marrow and liver [53].

### 5.6. Blood and Other Cancers

Additionally, curcumin is able to repress the growth of a variety of malignant cell types together with the lymphoma cells. The treatment of Burkitt's lymphoma cell lines with curcumin in combination with ionizing radiation (IR) indicates that curcumin application increases the sensitivity of lymphoma cells to IR-initiated apoptosis and improves G2/M phase arrest in the cell cycle [54]. Consequently, downregulation has been noted in the antiapoptotic Bcl-xL, cell cycle changing protein. Initiation of G2/M phase arrest (by curcumin) was found connected with an obvious reduction in the cyclin A, cyclin B, and cyclin-dependent kinase 1 protein expression. Also, apoptosis induction by curcumin is escorted with upregulation of the Bax protein expression and downregulation of the Bcl-2 protein amount which results in mitochondria dysfunction. Consequently, it leads to cytochrome c release and sequential activation of caspase-9 and caspase-3 in the nasopharyngeal carcinoma-TW 076 cells. Therefore, it seems that mitochondria and apoptosis-inducing factor caspase-3-dependent pathways are the fundamental figures in G2/M phase arrest and cell apoptosis by curcumin [55]. Curcumin also appreciably reduced the nuclear translocation of p65 and cytoplasmic I*κ*B*α* dilapidation. Survivin and hexokinase II have a trend to decrease by the curcumin pretreatment. Combined treatment of curcumin and L-asparaginase (L-ASP) initiates the apoptosis by activating various members of cysteine proteases (caspase-8 and caspase-9/3) along with activating phase-I detoxification system. Curcumin acts synergistically with L-ASP in patients suffering from blood and bone marrow cancer [56–58]. Moreover, curcumin drastically decreases the castrate-resistant disease and human leiomyosarcoma cell lines propagation and interrupts the cell growth of uterine leiomyosarcoma by targeting the AKT-mammalian target of rapamycin pathway for reticence [57, 59].

Curcumin in appreciable amounts decreases the T cells, while a small amount of curcumin enhances the T cells extracted from mice carrying 3LL tumor. Hence, amplified CD8+ T cells showed improvement in IFN-*γ* discharge and propagation particularly against 3LL tumor cells; all of these result in accomplishment of tumor inhibiting ability [60, 61].

The study regarding antiproliferative actions of turmeric components on human cancer cell lines including MDA-MB-231, MCF-7, and HepG2 and immunomodulatory actions of turmerones on mononuclear cells of human blood indicated that the alpha-turmerone and curcuminoids appreciably suppress the production of cancer cells. Improvement in the propagation of peripheral blood mononuclear cells and the makeup of cytokine has been noted after the application of alpha-turmerone and aromatic-turmerone [62].

As mentioned in the introduction, curcumin possesses numerous pharmacological properties but its reduced solubility in water restricts its clinical use. Therefore, preparation of curcumin into nanocarrier systems enhances its penetration into the tissues. For instance, curcumin-loaded nanocapsules markedly decrease the tumor volume [63].

## 6. Antioxidant and Anti-Inflammatory Activity

Recently, especial attention has been paid to turmeric due to its antioxidant activities which are carried through direct scavenging of oxygen radicals and stimulating antioxidant responses by nuclear factor erythroid 2-related factor 2 (Nrf2) activation. That feature, besides the favorable outcomes on the endothelial function and the inflammatory state of the tissue and plasma, indicated that it was helpful for the treatment of diabetic microangiopathy potentially [63]. The bioactive components present in turmeric volatile oil include tumerone that is reported to be effective against carcinogenesis. The previous investigation indicated that turmeric had distinctive antioxidant potential [64]. Curcumin removed turmeric oleoresin which is the resource material for the production of curcumin and contains oil, resin, and nonextractable curcumin. During a lab study, various fractions and turmeric oil exhibited considerable antimutagenic and antioxidant ability [28]. Curcumin at a concentration of 200 mg/kg body weight of female Wistar rats appreciably reduced the oxidative damage in the hippocampus of rats when exposed to the organophosphate pesticides parathion; thus, it is an alternative to prevent neurodegenerative damage after pesticide exposure [65].

In addition, turmeric extract has been reported to possess strong antioxidant activity, as shown by ABTS and DPPH tests. The antioxidant ability of turmeric resulted in decreased levels of prostaglandin E2 (as a marker of oxidative stress) in HepG2 cells [66]. Curcumin is also helpful in increasing the lifespan of *Caenorhabditis elegans* by reducing the intracellular ROS and lipofuscin during aging [67].

Previous research conducted to examine the ability of turmeric to protect against lead-induced harm to the hippocampal cells of male Wistar rats demonstrated that it extensively prevented lipid peroxidation due to the exposure of toxic heavy metals. The reaction between curcumin and metals (cadmium and lead) resulted in formation of complex compounds that showed the effectiveness of curcumin in binding the metals [68].

It should be noted that curcumin alters the neuroendocrine role of the central nervous system, thus reducing chronic stress-induced disorders. Curcumin treatments mitigate the anxiety reaction because of its ability to prevent the neurons through the modification of nitric oxide making and brain-derived neurotrophic factor (BDNF) expression [69].

In fact, turmeric holds significant anti-inflammatory properties, mainly via Wnt/*β*‐catenin, nuclear factor-kappa B (NF-*κ*B) (possibly by blockage of myeloid differentiation primary response 88 and toll-like receptor 4/NF-*κ*B signal [70] and downregulating mRNA expression of NF-*κ*B-p65 [71]), and mitogen-activated protein kinases pathways, and also by epigenetic modulatory role and redox regulation [72]. Another recent research showed the inhibitory effect of curcumin on NACHT, LRR, and PYD domain-containing protein 3 inflammasome activation [73]. Furthermore, it seems that signal transducer and activators of peroxisome proliferator-activated receptor-*γ* (PPAR*γ*) and transcription-3 are modulated by the turmeric supplementation [74]. A recent study explained its anti-inflammatory action through the phosphorylation repression of the I*κ*B kinase *α* and *β* and c-Jun N-terminal kinase, too [75].

Some other scientists explained that obstruction in T-cell-activation-induced Ca^2+^ mobilization (IC50 = ∼12.5 *μ*M) is another possible mechanism explaining anti-inflammatory and immunosuppressive ability of curcumin. They are also of the view that the same mechanism prevents nuclear factor of activated T-cell (NFAT) activation and NFAT-regulated cytokine expression. Moreover, curcumin can synergize with CsA to improve immunosuppressive action. In addition, because Ca^2+^ is also the vital messenger for the TCR-induced NF-*κ*B signaling pathway, it provides another mechanism by which curcumin suppresses the NF-*κ*B activation [76]. A systematic review revealed that curcumin can make its anti-inflammatory effect by modifying several proinflammatory cytokines (e.g., TNF-*α*, IL-6, and IL-8) in a physically active cohort [77]. The mentioned action was also observed in an in vitro model of intestinal inflammation. Besides it, the protective effect of turmeric on the intestinal epithelium may be promising for patients with inflammatory bowel disease [78]. Also, it has been shown that turmeric significantly lowers the level of high-sensitivity C-reactive protein (as an acute-phase protein) in a number of clinical trials [79, 80].

Furthermore, the role of curcumin in human ectopic endometriotic stromal cells secluded from women with endometriosis has been investigated. It was noted that the treatment of endometriotic stromal cells with curcumin indicated noticeable repression of mRNA expression of ICAM-1 and VCAM-1. Curcumin also drastically diminishes the TNF-*α*-initiated cell surface and emergence of ICAM-1 and VCAM-1. Moreover, the application of curcumin on the endometriotic stromal cells clearly reduces the TNF-*α*-initiated discharge of IL-6, IL-8, and monocyte chemoattractant protein-1 (MCP-1). Besides, curcumin subdues the commencement of transcription factor NF-*κ*B in human endometriotic stromal cells [40].

It has been studied that curcumin drastically reduces the pancreas injury and significantly improves the expression of PPAR*γ*. Curcumin application results in modulation of cytokine TNF-*α* release that could be a possible justification for its ability to attenuate the pancreas injury. These effects collectively lead to the upregulation of PPAR*γ* and downregulation of NF-*κ*B [81, 82]. Spinal cord injury (SCI) including primary SCI and secondary SCI is due to inflammatory bursts. However, primary SCI is the consequence of direct injury to the spinal cord, but secondary injury results from subsequent edema and ischemia that lead to the activation of proinflammatory cytokines. These cytokines result in the activation of NF-*κ*B and create hindrance in spinal cord reinnervation because of gliosis. Turmeric can inhibit the NF-*κ*B, and epidural administration of curcumin results in increased recovery from SCI without any side effects. For that reason, curcumin treatment may decipher a new treatment for humans with SCI [83].

In the nutshell, conclusive scientific evidence is available, reporting the effectiveness of turmeric and its components/fraction to treat the maladies characterized by oxidation and inflammation, thus warranting its use in dietary regimens.

## 7. Antimicrobial Effects

Turmeric may be an alternative antimicrobial agent against fatal bacterial infections [84]. The utilization of essential oil of turmeric leaves significantly inhibits fungal growth, as well as aflatoxins B1 and G1 production. Although curcumin is a very active agent, its reduced aqueous solubility hinders its applications. The nanocurcumin actually performs its antibacterial action by completely breaking the cell wall, leading to cell death [85].

Curcumin antibacterial activity against multidrug-resistant *Acinetobacter baumannii* noticeably increases in the presence of epigallocatechin gallate (EGCG). The combination of EGCG and curcumin can be used in medicine to avoid or control *Acinetobacter baumannii* infections [86].

Elimination of *Acanthamoeba castellanii* (as the causative agent for *Acanthamoeba* keratitis and granulomatous amoebic encephalitis) is complicated as the amoebas encyst makes it defiant to antiamoebic drugs. Amoebicidal activity of ethanol extracts of variant plants including peanut, sea daffodil, and turmeric was assessed on *Acanthamoeba castellanii* cysts. The results confirmed the inhibitory effect of the extracts on the duplication of *Acanthamoeba* cysts. However, the effect was time- and dose-dependent [87].

In addition, turmeric mouthwash can be successfully used as an adjunct method to mechanical plaque managing measures for the prevention of plaque and gingivitis. It should be noted that turmeric mouthwash results in significant diminution in total microbial count [88].

Turmeric hinders *Bacillus subtilis* and *Escherichia coli* growth by restraining filamenting temperature-sensitive mutant Z (FtsZ) (cytoskeletal protein) assembly through repressing the FtsZ polymerization [89]. Additionally, Khalafalla and his team estimated the anticoccidial activity of curcumin. They reported the sporozoites disconfiguration owing to inflammation and cell membrane corrugations [90]. Curcumin also dose-dependently reduces infectivity and cell proliferation. It dramatically represses the cytotoxicity of *Vibrio vulnificus* to HeLa cells by inhibiting *V. vulnificus* growth. Curcumin subdues both bacterial adhesion and RTX toxin binding to the host cells. Curcumin also inhibits the host cell rounding and actin aggregation. Moreover, curcumin reduces the *V. vulnificus*-induced NF-*κ*B translocation in the HeLa cells [91].

It should be noted that curcumin, as the main ingredient of turmeric, has a wide-ranging antiviral activity [92]. For instance, there are several studies on its different mechanisms against human immunodeficiency viruses (HIVs). Curcumin has been shown to inhibit HIV-1 integrase [93]. Moreover, this polyphenol and its analogues can inhibit the infection and replication of viral genes. HIV protease and HIV associated kinases (e.g., tyrosine kinase) are inhibited by them. Also, curcumin has synergistic effect with biomedicine drugs [94].

It should be noted that curcumin inhibits the Apurinic/apyrimidinic endonuclease-1 redox function. Therefore, a wide variety of genes and pathways are affected. It has been found that curcumin can inhibit Kaposi's sarcoma-associated herpesvirus replication and then control the consequent pathologic processes (e.g., angiogenesis) [95].

There are a number of researches on anti-influenza activity of biological constituents of turmeric [96]. It can fight influenza-A virus (IAV) via the inhibition of its adsorption and replication [97]. Moreover, curcumin can balance the immune system response through product inhibition of local inflammatory cytokines in IAV infection. Furthermore, it has modulatory effect on NF-*κ*B signaling in macrophages. In conclusion, it can play protective and ameliorative role in IAV-associated lung injury [98].

## 8. Neuroprotective Effects

Plant-derived components have been reported to not only provide neuroprotection but also manage biochemical pathways linked with symptoms of neurodegenerative disorders that comprise cognitive impairments, energy loss/fatigue, mood changes, and anxiety. Furthermore, it has neurogenic potentials which seems to be done via the stimulation of neural stem cell proliferation and differentiation [99]. Plants and their products having neuroprotective effects might be a novel therapeutic approach to treat Parkinson's disease (PD) [100, 101]. Aggregation of *α*-synuclein protein at high temperatures is a possible mechanism that leads to PD. Physiological pathway concerned in aggregation is unfolding the tetramer to kinetically trapped monomers that further arrange them, thus leading to the formation of fibrillar Lewy bodies. Therefore, avoiding reassociation of the monomers might be a helpful therapeutic approach to the prevention of PD. In a few research studies, investigators reported the ability of turmeric's ingredient to bind to *α*-synuclein, thus preventing the protein from aggregating and consequently escalating the rearrangement rate into a faster regime [102, 103]. However, most of the research studies highlighting neuroprotective possessions of turmeric in PD relied on rat models with different study durations. Some of their results showed that turmeric stimulates the enzyme action of *γ*-glutamyl cysteine ligase and guarded against protein nitration and disintegration of the neurons in the brain [104].

The onset of oxidative stress is another concern with mitochondrial degeneration leading to PD. Turmeric improves the glutathione (GSH) synthesis in experimental models, thus reducing the free radical damage and indeed oxidative stress. Bioconjugates of curcumin can play a major role against oxidative stress in dopaminergic neuronal cells and improve neuroprotection [105]. Curcumin improves the BDNF, phosphor-tyrosine kinase B (TrkB), phosphor-extracellular signal-regulated kinase, and AKT. It has been theorized that the neuroprotection of curcumin might be mediated via BDNF/TrkB-MAPK/PI-3K-CREB signaling pathway [106].

Alzheimer's disease (AD) also includes chronic inflammatory responses linked with both brain injury and beta-amyloid related pathology. It has been shown that oxidative stress and distressed protein metabolism and their interaction are central to AD pathogenesis [107]. Turmeric extract might be a potential source for the prevention of AD [108]. The brains of AD patients experience numerous modifications (i.e., distraction of protein synthesis, protein deprivation, and imbalanced heat shock response (HSR)). The HSR is responsible for the protection of the cells from a variety of stresses. Curcumin utilization could be a dietetic approach used to decrease oxidative injury and amyloid pathology connected with AD; thus, it could be a powerful tool in the prevention of AD [107, 109].

The encapsulation of bioactive components is gaining the attention of the researchers due to their claimed better efficacy. In a study, researchers evaluated the cytotoxicity of encapsulated nanoparticles of curcumin (Nps-Cur). They were of the view that human neuroblastoma SK-N-SH cells exposed to Nps-Cur showed the least signs of toxicity. Therefore, Nps-Cur can be a good option to provide neuroprotection to the patients suffering from AD [110].

Cerebral ischemia is related to the amplified TdT-mediated dUTP nick-end labeling (TUNEL) positive cells in brain sectors representing DNA disintegration. Treatment with curcuma oil may be helpful to reduce nitric oxide synthase (NOS) isoforms and noteworthy decline in the number of apoptotic cells during cerebral ischemia [111]. Also, curcumin reduces the expression of lipopolysaccharide-induced chemokine CCL2 mRNA and protein in C6 cells [112]. The water-soluble curcumin formulations (50–200 mg/kg) reduce the serenity period and enhance serotonin and dopamine levels in the brain tissues. As a result, these curcumin preparations might be a new opportunity for the modifications in the neurotransmitters and treatment of mental depression [113].

Finally, there are several studies on turmeric's neuroprotective effect in traumatic brain injury. Results showed this effect by decreased oxidative stress and cerebral edema, rise in BDNF level, protection of synaptic proteins and mitochondria, and microglial activation. Also, it has been shown that it can decrease in IL-6, TNF-*α*, IL-1*β*, and MCP-1 and improve toll-like receptor 4 and aquaporin-4 expression [114]. Additionally, the activation of the Nrf2 pathway is one of the most important mechanisms regarding this action [115].

### 8.1. Effects on Metabolic Syndrome, Related Disorders, and Cardiovascular Diseases

There are several recent systematic reviews on the effects of curcumin in patients with metabolic syndrome and related disorders. As the results of meta-analysis showed, there is a significant improvement regarding a wide variety of indices such as fasting blood sugar, insulin resistance index (i.e., HOMA-IR), glycated hemoglobin (i.e., HbA1c), triglycerides, leptin, adiponectin, total cholesterol, diastolic blood pressure, body mass index, and weight. However, it seems that its consumption is not associated with a significant change in systolic blood pressure and low-density lipoprotein cholesterol level, and hip ratio. It should be noted that there are discrepancies regarding its effect on high-density lipoprotein cholesterol and waist circumference [116–118].

In fact, the supplementation of turmeric extract exerts antiobesity effects by controlling the body weight, fat mass, serum lipids, and hepatic lipids. Furthermore, protein kinase A pathway-activated lipolysis might be another possible path [119]. The supplementation of lecithinized formulation of curcumin to diabetic patients (at a dose of 1 g/day) pointed out a decrease in the skin flux at the surface of the foot, improvement in microangiopathy, momentous reduction in the edema score, and a subsequent progress in the venoarteriolar reaction [120].

Furthermore, turmeric extract at the dose of 100–300 mg/kg of the body weight of hypercholesterolemic subjects showed considerable progress in vaso-relaxation. The levels of the antioxidant enzymes including superoxide dismutase and glutathione peroxides increased in subjects fed on diets containing turmeric extract, too [121].

The separation of curcuminoids also results in turmeric spent oleoresin (SOT) that is another fraction reported for some health benefits. SOT enriched with curcuminoids (17.5%) can efficiently restrain angiotensin-converting enzyme and low-density lipoprotein oxidation. Likewise, curcuminoid-enriched fraction can lessen the risk of hypertension and cardiovascular diseases [122].

Different spices, including turmeric, have an inevitable role in the prevention and treatment of cardiovascular diseases. Modulating migratory, proliferative, and hypertrophic pathways, prohypertrophic signaling, and proliferation abilities of cardiac fibroblasts are some of the mechanistic aspects of its cardiovascular protection. In addition, it can make a balance between extracellular matrix components' synthesis and degradation [123–125].

There are so many preclinical researches on turmeric use for a wide variety of cardiovascular diseases such as myocardial infarction, heart failure, cardiac hypertrophy, atherosclerosis, stroke, abdominal aortic aneurysm, drug-induced cardiotoxicity, and cardiomyopathy, and diabetes mellitus related cardiovascular problems. Also, there are a number of clinical trials about the efficacy of curcumin on cardiovascular risk factors [126–128].

Furthermore, turmeric and curcumin have well-known antidotal effects against natural and chemical agents-induced cardiovascular toxicity. Scientific reports suggested their beneficial roles in streptozotocin, methotrexate, doxorubicin, ciclosporin A, cadmium, isoproterenol, hydrogen peroxide, nicotine, diesel exhaust particle, tert-butyl hydroperoxide, and cyclophosphamide toxicities [129, 130].

## 9. Safety and Toxicity

Turmeric and its constituents were examined in many researches for their safety through in vitro studies, animal studies, and clinical trials. According to a comprehensive review on this subject, the administration of standardized powder/extract of turmeric and curcumin via oral route revealed no significant side effects or toxicities to animals. In addition, cell culture studies showed that “*curcumin has antiproliferative effect in normal cells and can reduce cell viability.*” However, there were no reports about mutagenicity and genotoxicity. It seems that oral use of turmeric and curcumin in human is safe, even at extraordinary doses. Itching, tongue redness, tachycardia, and gastrointestinal complaints (e.g., flatulence, diarrhea, nausea, and constipation) were reported in a small proportion of cases. It should be noted that there are several problems regarding bioavailability of oral curcumin. However, its intravenous formulations have a greater absorption. Therefore, intravenous curcumin should be administered at lower doses than oral use [131].

It should be noted that curcumin may cause some kinds of pharmacokinetic alteration of cardiovascular medications, antibiotics, antidepressant agents, chemotherapeutic drugs, anticoagulants, and antihistamines. Therefore, its concomitant use with some conventional drugs should be done cautiously [132].

Oral use of turmeric and curcumin should be considered safe during pregnancy, according to animal studies. However, there is a report stating that oral curcumin about 1000 mg/kg body weight can cause a slight reduction of weight gain in F2 generation chicks [131].

Regarding the application of drug delivery technologies for improving turmeric bioavailability, safety of these modern formulations should be discussed. For instance, using carriers or surfactants as a bioavailability improving strategy may cause products to be toxic [133]. Also, some of the inorganic metal nanoparticles (e.g., gold-curcumin nanoparticles) are highly toxic [84].

However, most of these novel products seem to be safe. Solid lipid curcumin particle formulation showed no adverse effects in patients with osteosarcoma and healthy peoples [134]. Poly(N-isopropylacrylamide) delivery system, as another novel system which transports curcuminoids to the brain nasally, showed no toxicity [135]. Dipeptide nanoparticles of curcumin are safe, too. Dipeptide is synthesized from amino acids *α*,*β*–dehydro phenylalanine and methionine, which are safe and capable of being decomposed in nature [136]. New curcumin analogues are another recently used option in medicine. According to in vivo studies, they showed no toxicities [137]. Moreover, nanoparticles of curcumin-loaded human serum albumin showed no toxicity during intravenous use in tumor xenograft HCT116 models [138]. In some cases, modernized formulation of curcumin seems to be safer than conventional ones. Results of a study on a novel intravenously injectable curcumin revealed that rabbits in curcumin nanosuspension group developed local irritation and phlebitis risks and erythrocyte hemolysis lesser than those in curcumin solution group [139].

## 10. Conclusion

The most widely used part of turmeric is its root, comprising loads of phytochemicals, vitamins, and minerals, quite beneficial for the cure of various human diseases. It seems that turmeric is a generally safe medicinal herb. However, its use during pregnancy and lactation and in patients with hepatic and renal failure should be assessed critically. Many products are extracted from turmeric. Its most active component, curcumin, followed by other significant components including curcuminoids atlantone, dimethoxycurcumin, diarylheptanoids, tumerone, and flavonoid curcumin (diferuloylmethane) has antimicrobial, anti-inflammatory, and antioxidant properties that provide its protective effect against different types of cellular injury. Moreover, turmeric and its constituents not only provide neuroprotection but also modulate the pathology of neurologic diseases like Parkinson's and Alzheimer's diseases. It has also been well documented to be an effective tool against various kinds of cancers. Furthermore, there are several researches on its effects against metabolic syndrome. To sum up, it could be concluded that turmeric and its components are recommended in dietary regimens to combat a wide variety of diseases. Further researches are necessitated for better understanding and judgment on its uses in clinical practice. Furthermore, regarding its constituents' bioavailability and drug delivery systems, developing modern formulations (e.g., nanoparticles, liposomes, and microspheres) and assessing their efficacy are suggested.

## Figures and Tables

**Table 1:** Nutritional composition of turmeric.

Principle constituents	Nutrient value (kcal)	Percentage of RDA (%)
Energy	354	17
Carbohydrates	64.9	50
Total fat	9.88	33
Protein	7.83	14
Cholesterol	0	0
Dietary fiber	21	52.5
Vitamins		
Pyridoxine	1.80	138
Folates	39	10
Niacin	5.140	32
Riboflavin	0.233	18
Vitamin A	0	0
Vitamin C	25.9	43
Vitamin E	3.10	21
Vitamin K	13.4	11
Electrolytes		
Potassium	2525	54
Sodium	38	2.5
Minerals		
Manganese	7.83	340
Calcium	183	18
Copper	603	67
Iron	41.42	517
Magnesium	193	48
Phosphorus	268	38
Zinc	4.35	39.5

**Table 2:** The main products of turmeric, their descriptions, and uses.

Product name	Description	Uses
Whole rhizome (dried form)	Appearance: orange-brown, red-yellow, or pale yellow	Medicinal purposes
	Chemical composition: it may contain 3–15% curcuminoids, and 1.5 to 5% essential oils	
	Preparation: finger rhizomes and mother rhizomes are generally boiled, separately for about 40–60 min, under slightly alkaline conditions. It should be followed by sun-drying for 10–15 days to diminish the moisture content about 10%	

Ground turmeric	Appearance: either yellow or red-yellow in color	Used as a spice, dye, medicine, and as a dietary supplement
	Chemical composition: the main active ingredients (i.e., curcuminoids and essential oils) may lessen during the process and also by exposure to light. It is necessary to pack the powder in a UV protective container	
	Preparation: dried finger rhizomes are grounded to produce its powder	

Turmeric oil	Appearance: yellow to brown oil	Used as spice, medicine, and dietary supplement
	Chemical composition: essential oils from the leaves are usually dominated by monoterpenes. Rhizomes oil mainly contains sesquiterpenes	
	Preparation: extract procured from dried rhizomes or leaves by steam distillation or supercritical CO_2_ extraction	

Turmeric oleoresins	Appearance: dark yellow, reddish-brown viscous fluid	Used as a food coloring, medicine, and dietary supplement
	Chemical composition: they consist of up to 25% essential oil and 37–55% curcuminoids	
	Preparation: extract from dried rhizomes by solvent extraction with organic solvents (acetone, dichloromethane, 1,2-dichloroethane, methanol, ethanol, isopropanol, and light petroleum (hexanes)) or by the application of supercritical CO_2_ extraction	

Curcumin	Appearance: crystalline powder of yellow to orange-red color	Used as medicine and dietary supplement
	Chemical composition: a mixture of curcumin and its bisdemethoxy- and demethoxy- derivatives (no fixed proportions). The three major curcuminoids may occupy 90% of the whole proportion. Oils and resins may be the minority of composition	
	Preparation: it is obtained by solvent extraction from ground turmeric rhizomes followed by the purification of the extract through the crystallization process	
	Organic solvents used for extraction are acetone, carbon dioxide, ethanol, ethyl acetate, hexane, methanol, and isopropanol	

## Abbreviations

C. Longa: Curcuma longa
TNF-*α*: Tumor necrosis factor alpha
ABTS: 2,2′-Azino-bis (3-ethylbenzothiazoline-6-sulfonic acid)
AD: Alzheimer's disease
BDNF: Brain-derived neurotrophic factor
CDC27: Cell division cycle 27
COX-2: Cyclooxygenase 2
CRC: Colorectal cancer
NF-*κ*B: Nuclear factor-kappa B
DPPH: 2,2-Diphenyl-1-picrylhydrazyl
EGCG: Epigallocatechin gallate
GSH: Glutathione
HSR: Heat shock response
ICAM-1: Intercellular Adhesion Molecule 1
IGF-1: Insulin-like growth factor-1
L-ASP: L-Asparaginase
MCP-1: Monocyte chemoattractant protein 1
NFAT: Nuclear factor of activated T cells
NF-*κ*B: Nuclear factor-kappa B
NOS: Nitric oxide synthase
Nps-Cur: Encapsulated nanoparticles of curcumin
Nrf2: Nuclear factor erythroid 2-related factor 2
PD: Parkinson's disease
PPAR*γ*: Peroxisome proliferator-activated receptor-*γ*
RANKL: Receptor activator of nuclear factor-kappa B ligand
ROS: Reactive oxygen species
SCI: Spinal cord injury
SOT: Turmeric spent oleoresin
TrkB: Tyrosine kinase B
TUNEL: TdT-mediated dUTP nick-end labeling
VCAM-1: Vascular cell adhesion molecule 1
STAT3: Signal transducer and activator of transcription 3
IR: Ionizing radiation
IL: Interleukin
FtsZ: Filamenting temperature-sensitive mutant Z
HIV: Human immunodeficiency viruses
IAV: Influenza-A virus.
